# The impact of surgical staging in patients with colorectal peritoneal metastases scheduled for CRS-HIPEC

**DOI:** 10.1515/pp-2024-0013

**Published:** 2025-04-28

**Authors:** Lana Ghanipour, Johan Wallin, Peter Cashin, Wilhelm Graf

**Affiliations:** Department of Surgical Sciences, Uppsala University, Uppsala, Sweden

**Keywords:** CRS-HIPEC, staging laparoscopy, colorectal peritoneal metastases

## Abstract

**Objectives:**

Surgical staging procedures are used to select patients with peritoneal metastases for surgery. We aimed to evaluate the impact of surgical staging procedures and the risk of abdominal wall recurrences in patients with peritoneal metastases scheduled for cytoreductive surgery and hyperthermic intraperitoneal chemotherapy (CRS-HIPEC).

**Methods:**

Data were collected from a prospective maintained HIPEC register January 2012–December 2019. Patients with colorectal peritoneal metastases were included. Information about surgical staging procedures was registered. Results were then compared with those registered at definite CRS-HIPEC surgery and survival was analysed in relation to surgical staging procedures.

**Results:**

In total, 138 patients were included, of whom 32 had undergone a surgical staging procedure before CRS-HIPEC. Median overall survival in the surgical staging group was 1.89 years and in the non-staging group 3.07 years (p=0.060). In the surgical staging group, eight patients developed abdominal wall recurrences (25 %) compared with three (3 %) in the non-staged group. Eight staged patients (25 %) were considered inoperable at definite surgery (open-close). PCI score (p<0.001) was higher at definite surgery in patients who had undergone a staging laparoscopy. Factors associated with shorter overall survival in multivariate analysis were: open and close, PCI ≥21 and presence of signet ring cells. However, a staging procedure was not associated with a shorter overall survival.

**Conclusions:**

Surgical staging procedures is a valuable assessment of inoperability, though at the expense of more frequent abdominal wall recurrences. Imaging-based strategies may provide useful insights into whether surgical staging is the most effective approach for patient selection.

## Introduction

The peritoneum is the third most common location of metastases from colorectal cancer with an incidence of around 8 %, occurring equally in a synchronous or metachronous situation [1]. The standard treatment of isolated peritoneal metastases is cytoreductive surgery (CRS) and hyperthermic intraperitoneal chemotherapy (HIPEC) [2], 3]. However, the prognosis is largely dependent on the extent of peritoneal spread as measured with the peritoneal cancer index (PCI) [4], 5]. Preoperative work up includes abdominal computed tomography in order to give a rough estimate of the extent of peritoneal growth, and in borderline cases, a surgical staging procedure, i.e. laparoscopy or laparotomy is used to more precisely assess the PCI [6], but few studies have evaluated the value of surgical staging procedures as a tool to select patients for CRS-HIPEC.

One limitation associated with diagnostic laparoscopy is incomplete preoperative surgical staging, which occurs in around 20 % of the cases, mostly due to adhesions after previous surgery. This results in incomplete visualization of the abdominal regions and thereby limits the selection of patients eligible for CRS-HIPEC [7]. Furthermore, tumour cell entrapment resulting in port site metastases might occur after laparoscopy. However, laparoscopy is considered a safe and feasible procedure for staging peritoneal metastases and negligible surgical complications have been reported [8]. Staging laparotomy is a more invasive procedure and is also associated with a risk of tumour entrapment in the abdominal wall incision.

Our study aimed to assess the prognostic impact of surgical staging procedures in patients with peritoneal metastases scheduled for CRS-HIPEC with respect to overall and recurrence-free survival, frequency of abdominal wall metastases, and proportion of inoperability (open and close). In the assessment, staging procedures performed on purpose were considered separately. A secondary aim was to compare the PCI at the staging procedure with that recorded at the definite CRS-HIPEC procedure.

## Methods

### Patients and data collection

Patients with a histopathologically verified colorectal cancer with peritoneal metastases scheduled for a primary CRS and HIPEC procedure were included. Data were collected from patient records and a prospectively maintained HIPEC register. We registered: age, gender, location of primary tumour, type of treatment (CRS-HIPEC, CRS only, debulking or laparotomy only because of inoperability, hereafter named open-close), HIPEC-regimen, peritoneal cancer index (PCI), completeness of cytoreduction (CC) score [9], [10], [11], neoadjuvant treatment, surgical staging procedure with laparotomy or laparoscopy or alternatively staging of the peritoneal metastases during elective or emergency surgery, resection of abdominal wall at definite CRS-HIPEC, postoperative histopathology in peritoneal specimens and the abdominal wall, and presence of signet ring cells.

The surgical staging group were categorised into four groups; discovery of peritoneal metastases during emergency surgery mostly due to intestinal obstruction; during primary tumour surgery with unexpected finding of peritoneal metastases; decisions made during multidisciplinary team meeting for a surgical staging procedure requested by the HIPEC centre; and lastly findings of peritoneal metastases during other procedures such as cholecystectomy, liver or hernia surgery.

**Figure 1:**
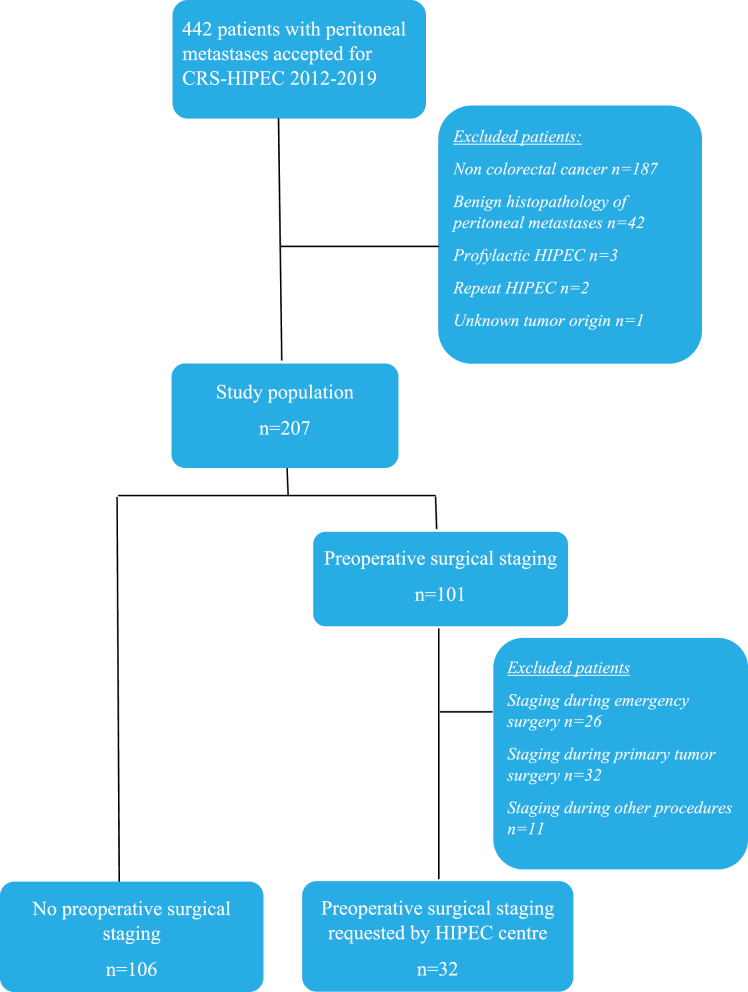
Flow-chart of the study cohort.

To ensure a more homogeneous cohort and facilitate comparison between staged and non-staged patients, only patients who had undergone a surgical staging procedure requested by the HIPEC-centre (n=32) were included in this study, yielding a total of 138 patients (Figure 1). The staging group consisted of patients who had undergone staging at a referral hospital with expertise in colorectal surgery, though the exact experience of the referral hospital experience was not recorded.

Surgical staging procedure were requested by the HIPEC centre for patients deemed suitable for staging based on radiological finding, including those without extensive adhesions but with radiological suspicion of significant tumor growth, possible small bowel involvement or advanced tumor growth in regions involving pancreas, stomach or hepatic pedicle.

The surgical staging procedure was performed either as laparoscopy or mini-laparotomy, before or after neoadjuvant therapy, depending on the radiological findings. Although the radiological PCI score is not routinely documented at our centre, staging was primarily indicated for cases where CT imaging suggested a PCI score greater than 15.

All patients were discussed in a multidisciplinary team meeting before being considered eligible for CRS-HIPEC. Patients with good performance status, age <80 years, few comorbidities or limited tumor extent on the CT-scan were considered CRS-HIPEC.

Information about previous staging laparoscopy or laparotomy was registered from referral notes, including PCI score at the staging procedure and whether there was small bowel involvement.

Information on survival was obtained from the Swedish Population Register and all observations were censored in December 2020.

The study was approved by Uppsala County´s Ethics Committee, DnR 2013/203.

### Surgical procedure and HIPEC regimens

Surgical resections of peritoneal metastases were performed according to established clinical guidelines [12] and the extent of peritoneal tumours was assessed after abdominal exploration using the PCI score [11]. The outcome of CRS was categorized using the CC score [10]. Patients with extensive peritoneal metastases, in whom there was no possibility of achieving a complete macroscopic resection were considered inoperable (open-close or debulking). In open-close cases, only palliative procedures like intestinal resection, stoma or by-pass in case of obstruction, were performed. Limited and intentionally not radical tumour resection, i.e., resection of the greater omentum or ovaries, was categorized as a debulking procedure.

Criteria for abdominal wall or port site scar resection for histopathology was if the preoperative radiological or intraoperative assessment indicated malignant infiltration in the abdominal wall scars or in case of suspected malignant infiltration during exploration. The abdominal wall incision and port site scars were resected initially during the access to intraperitoneal cavity and the resections were performed with approximately 10 mm margin. Otherwise, the abdominal wall scars were left without resection if no sighs of abdominal wall or port site recurrence were present.

The HIPEC treatment was given according to the Coliseum method [13] with intraabdominal administration of either Oxaliplatin (460 mg/m^2^), Irinotecan (460 mg/m^2^) or Mitomycin C (30 mg/m^2^) for a duration of either 30 min (Oxaliplatin, Irinotecan) or 90 min (Mitomycin C). Before administration of oxaliplatin or irinotecan intraperitoneally, a single dose of intravenous 5-FU (400 mg/m^2^) was given, followed by folinic acid, 60 mg/m^2^, 30 min before the administration of HIPEC. Intraabdominal temperature was maintained at 41–43 ºC.

### Statistical analysis

Pearson´s Chi^2^ test was used to compare differences in proportions between the groups and the Mann-Whitney U test was used to compare differences in continuous variables. Overall survival was measured from the date of surgery to the date of death from any cause, and time to recurrence was defined as the interval from the date of surgery to the date of any documented recurrence. The univariate and multivariate Cox proportional hazard regression model was used to assess the relationship between clinicopathological variables and overall and recurrence-free survival in patients with CC-score 0. Log-rank test was used to analyse differences in median overall survival between the surgical staging group and no surgical staging group. Logistic regression was used to estimate the odds ratio (OR) with 95 % confidence intervals (CI) for abdominal wall recurrence. Time was censored at the end of study period (December 2020). For statistical analyses, SPSS version 27 (SPSS Inc. Chicago, Ill. USA) was used and statistical significance was set at p<0.05.

## Results

### Patient characteristics

A total of 442 patients were scheduled for CRS-HIPEC at Uppsala University Hospital from June 2012 to December 2019. One hundred and eight were low grade appendiceal tumours; 29 were appendiceal adenocarcinoma; 42 did not have microscopically verified peritoneal metastases; 40 had other primary tumour origins; three were prophylactic HIPEC; two were repeat CRS and HIPEC, and one patient had an exploration due to unknown tumour origin, leaving 220 patients in the cohort.

A preoperative staging procedure with laparoscopy or laparotomy before referral to the HIPEC centre was performed in 101 of the patients, of whom 32 (32 %) had undergone a separate diagnostic surgical staging procedure requested by the HIPEC centre in borderline cases. Staging during emergency surgery was performed in 26 patients (26 %) and in 32 patients (32 %) staging was performed during intended primary tumour surgery which was aborted after the unexpected finding of peritoneal metastases. In 11 patients (11 %), staging was accomplished during other procedures such as gynaecological surgery (n=6), liver surgery (n=4), and hernia surgery (n=1).

Further analyses are based on patients who had undergone a surgical staging procedure requested from the HIPEC-centre (n=32) and those without a surgical staging procedure (n=106) (Figure 1). None of the patients in the surgical staging group had severe complications related to the surgical staging procedure prior CRS-HIPEC as indicated by their ability to undergo subsequent CRS-HIPEC.

The median age was 65 years (range 22–80), and 85 were women and 53 men. In 122 (88 %) of the patients, the primary tumour was located in the colon, and in 16 (12 %) in the rectum. During the staging procedure, the PCI score was registered in 13 patients (41 %). Reporting PCI scores occurred in the majority of cases during the final study period that in the beginning (Table 1).

**Table 1:** Descriptive statistics of the study cohort and comparison of patients’ characteristics in those who had undergone a surgical staging procedure requested by the HIPEC-centre before CRS-HIPEC vs. those without preoperative staging surgery.

Patient characteristics, n	Total cohort 138, %	Surgical staging n=32 (%)	No surgical staging n=106, %	p-Value
Age (median)				
Range	28–80			
≤65 years	73 (53)	20 (63)	53 (50)	0.214
≥ 66 years	65 (47)	12 (37)	53 (50)	
Gender				
Woman	85 (62)	20 (63)	65 (61)	0.904
Man	53 (38)	12 (37)	41 (39)	
Primary tumour				
Colon	122 (88)	32 (100)	90 (85)	0.019
Rectum	16 (12)	0 (0)	16 (15)	
Synchronous	51 (37)	19 (59)	32 (30)	0.003
Metachronous	87 (63)	13 (41)	74 (70)	
Neoadjuvant chemotherapy				
Yes	32 (23)	7 (22)	25 (24)	0.841
No	106 (77)	25 (78)	81 (76)	
Type of surgery				
CRS-HIPEC	99 (72)	23 (72)	76 (72)	0.211
CRS	14 (10)	1 (3)	13 (12)	
Open & close	25 (18)	8 (25)	17 (16)	
HIPEC regimen				
Oxaliplatin	76 (55)	21 (66)	55 (52)	0.289
Irinotecan	19 (14)	2 (6)	17 (16)	
Mitomycin C	4 (3)	0 (0)	4 (4)	
No HIPEC	39 (28)	9 (28)	30 (28)	
Surgical staging of PM				
Laparoscopy		27 (84)		
Laparotomy		5 (16)		
PCI score at definite CRS-HIPEC surgery				
Median	12	21	11	<0.001
Minimum	0	5	0	
Maximum	39	39	38	
Missing data	4	0	4	
CC score				
CC0	99 (72)	19 (59)	80 (75)	0.084
CC1	11 (8)	5 (16)	6 (6)	
CC2-3	25 (18)	8 (25)	17 (16)	
Not known	3 (2)	0 (0)	3 (3)	
Time interval from surgical staging to definite surgery, days				
Median		52		
Minimum		3		
Maximum		277		
Missing data		4		
Registration of PCI score during staging procedure				
Yes		13 (41)		
No		19 (59)		
Division of PCI score registration during staging procedure in time periods				
2012–2016		1		
2017–2019		12		
PCI score at surgical staging				
Median		9		
Minimum		2		
Maximum		21		
Missing data		19		
Presence of signet ring cells				
No	120 (87)	26 (81)	94 (89)	0.311
Yes	15 (11)	5 (16)	10 (9)	
Not known	3 (2)	1 (3)	2 (2)	
Abdominal wall histopathology				
Port site metastasis	8 (6)	8 (25)	0	
Abdominal wall scar metastasis	0 (0)	0 (0)	0	
Metachronous wall scar metastasis	3 (2)	0	3 (3)	
No metastasis in the abdominal wall at histopathology	25 (18)	21 (66)	4 (4)	
No resection of abdominal wall after previous surgical staging procedure or at definite surgery	102 (74)	3 (9)	99 (93)	
Histopathology of the abdominal wall specimens				
Malignant	11 (9)	8 (24)	3(3)	<0.001
Benign	127 (91)	24 (76)	103 (97)	
Overall survival (median; years)	2.43	1.89	3.07	0.060

Values in parentheses are percentages. Differences analysed using log-rank test.

Median time interval from the staging procedure to definite CRS-HIPEC surgery was 52 days in total (Table 1). In a separate analyses the median time interval to definite CRS-HIPEC showed 54 days for staging laparoscopy and 13 days for laparotomy (data not shown). CRS-HIPEC was completed in 99 (72 %) of the cases. Fourteen patients underwent CRS only, and 25 patients (18 %) were considered ineligible for CRS-HIPEC (open-close or debulking). Oxaliplatin was the most common HIPEC regimen and complete cytoreduction was achieved in 99 (72 %) (Table 1). Fourteen patients did not receive HIPEC, mainly because of comorbidity and intraoperative complications.

Neoadjuvant treatment was given to 22 % of the surgical staging group (Table 1), demonstrating that most of the patients in the cohort underwent upfront CRS-HIPEC.

### Surgical results

**Figure 2:**
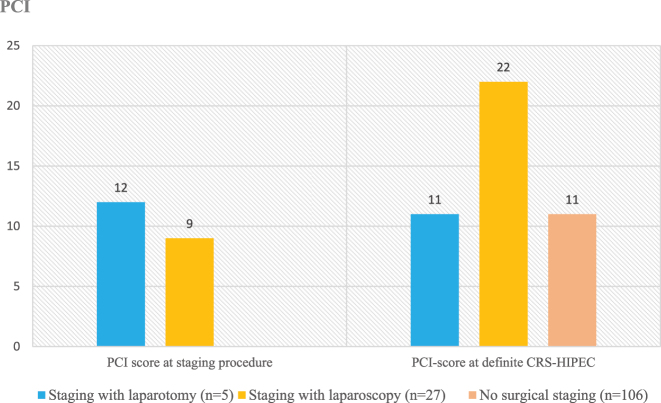
Distribution of median PCI-score registered at staging procedure and at definite CRS-HIPEC.

The intraoperative PCI score was higher in patients who had undergone a surgical staging procedure than in the group of patients without a staging procedure before CRS-HIPEC (p<0.001, Table 1). The median PCI score at definite surgery was higher compared to what was recorded at the surgical staging procedure, especially in those who had undergone a staging laparoscopy (Figure 2). The proportion of patients in whom radical surgery was performed was higher in the group without a staging procedure but the difference was not significant (p=0.084, Table 1).

In all, 36 patients underwent abdominal wall resection during definite CRS-HIPEC surgery, of whom 8 had verified port site recurrence and none with recurrences in the abdominal incision. In total, 25 patients had no histopathological malignancy in the abdominal wall and 102 patients had no clinical sign of abdominal wall recurrences detected during the definite CRS-HIPEC surgery, and therefore no abdominal wall resection was performed.

Abdominal wall recurrences were more common after a staging procedure (8/32, 25 %) compared to cases with no staging surgery (3/106, 3 %) before CRS-HIPEC (p<0.001, Table 1). The probability of abdominal wall recurrence was higher in the surgical staging group (OR 10.26; p=0.004 (Table 2).

**Table 2:** Logistic regression analysis of probability for abdominal wall recurrences.

Variable, n	OR	p-Value	95 % CI
Age			
≥ 66 years	10.1	0.051	0.99–103.4
Gender			
Woman	1.77	0.480	0.37–8.56
Metachronous	1.45	0.695	0.23–9.08
Neoadjuvant chemotherapy			
Yes	1.35	0.738	0.23–7.77
Surgical staging			
Yes	10.26	0.004	2.11–49.87
PCI score at definite CRS-HIPEC surgery			
>20	1.36	0.756	0.19–9.63
Presence of signet ring cells			
Yes	0.46	0.421	0.07–3.02

OR, odds ratio; CI, confidence interval.

### Survival analyses

Median overall survival in the surgical staging group was 1.89 years and in the non-staging group 3.07 years (Table 1). Factors associated with short overall survival in the multivariate Cox regression analyses were open & close (HR: 5.43, 95 % CI: 2.73–10.79), PCI score ≥21 (HR: 2.09, 95 % CI: 1.22–3.58), and presence of signet ring cells (HR: 2.20, 95 % CI: 1.21–4.02 (Table 3).

**Table 3:** Factors affecting overall survival in patients with peritoneal metastases from CRC scheduled for CRS-HIPEC.

	n	Univariate analysis HR (95 % CI)	p-Value	Multivariate analysis HR (95 % CI)	p-Value
Surgical staging procedure					
No	106	1 (Ref.)		1 (Ref.)	
Yes	32	1.59 (0.98–2.58)	0.062	1.44 (0.83–2.48)	0.195
Synchronous	51	1 (Ref.)		1 (Ref.)	
Metachronous	87	0.67 (0.43–1.06)	0.088	1.17 (0.67–2.04)	0.575
Primary tumour					
Colon	122	1 (Ref.)			
Rectum	16	0.56 (0.26–1.23)	0.147		
Neoadjuvant therapy					
No	106	1 (Ref.)			
Yes	32	0.88 (0.52–1.51)	0.650		
Definite surgery	114	1 (Ref.)		1 (Ref.)	
Open & close	24	6.29 (3.59–11.02)	<0.001	5.43 (2.73–10.79)	<0.001
PCI score at definite surgery					
≤20	96	1 (Ref.)		1 (Ref.)	
≥21	38	3.36 (2.12–5.34)	<0.001	2.09 (1.22–3.58)	0.007
Abdominal wall recurrence					
No	127	1 (Ref.)			
Yes	11	1.38 (0.66–2.87)	0.390		
Presence of signet ring cells					
No	120	1 (Ref.)		1 (Ref.)	
Yes	15	3.67 (2.05–6.58)	<0.001	2.20 (1.21–4.02)	0.010

Median overall survival for patients with abdominal wall recurrences was 1.94 years and 2.75 years for those without (log-rank p=0.387) and the recurrence free survival was 0.42 years for patients with abdominal wall recurrences and 0.69 years for those without (log-rank p=0.172). There was no increased risk of recurrence for those having abdominal wall malignant infiltration resected at CRS-HIPEC (p=0.180, Table 4). Instead, an increased risk of recurrence was associated with presence of signet ring cells (HR: 2.80, 95 % CI: 1.20–6.53) in the univariate analysis (Table 4).

**Table 4:** Factors affecting time to recurrence in patients with peritoneal metastases from CRC scheduled for CRS-HIPEC. Only cases that resulted in CC0 were included in the analyses.

	n	Univariate analysis HR (95 % CI)	p-Value	Multivariate analysis HR (95 % CI)	p-Value
Surgical staging procedure					
No	80	1 (Ref.)		1 (Ref.)	
Yes	19	1.13 (0.64–2.00)	0.679	0.95 (0.50–1.82)	0.880
Primary tumour					
Colon	87	1 (Ref.)		1 (Ref.)	
Rectum	12	0.98 (0.50–1.91)	0.952	0.99 (0.50–1.99)	0.987
Synchronous	32	1 (Ref.)		1 (Ref.)	
Metachronous	67	1.00 (0.61–1.63)	0.985	1.07 (0.63–1.85)	0.776
PCI score at definite surgery					
≤20	86	1 (Ref.)		1 (Ref.)	
≥21	13	1.23 (0.63–2.40)	0.544	0.75 (0.29–1.92)	0.546
Abdominal wall recurrence					
No	93	1 (Ref.)		1 (Ref.)	
Yes	6	1.78 (0.77–4.12)	0.180	1.13 (0.35–3.59)	0.839
Presence of signet ring cells					
No	91	1 (Ref.)		1 (Ref.)	
Yes	8	2.80 (1.20–6.53)	0.017	3.56 (0.92–13.7)	0.065

## Discussion

Our study evaluated the value of preoperative surgical staging in patients with peritoneal metastases from colorectal cancer eligible for CRS-HIPEC. This question is not only important for surgeons who specialize in CRS-HIPEC but also for surgeons referring patients to a CRS-HIPEC centre. Staging laparoscopy has long been considered a valuable tool for assessing the extent of peritoneal tumours and predicting whether complete cytoreduction is achievable [6]. We found that 25 % of the patients were inoperable at definite surgery after undergoing preoperative surgical staging, mainly due to widespread peritoneal metastases with a PCI >20. These findings align with those of Iversen et al. reported, who reported that 10 out of 27 patients were ineligible for CRS-HIPEC due to advanced tumour growth in difficult-to-assess regions, such as the pancreas, hepatic pedicle, and ureters, despite prior staging laparoscopy. These regions are challenging to evaluate laparoscopically, highlighting the limitations of staging laparoscopy in certain cases [7].

At our center, 72 % of patients completed CRS-HIPEC, while 3 % underwent CRS only after a preoperative staging procedure requested by the HIPEC-center. These results are in agreement with the results of Iversen et al. Over the past decade, we have routinely employed laparoscopy in the preoperative diagnostic assessment of selected patients with peritoneal metastases from colorectal and appendiceal cancer. This is particularly useful when radiological staging is uncertain or when assessing small bowel involvement is crucial for predicting the possibility of complete cytoreduction. Patient selection for surgical staging procedure is typically driven by the limitations of radiological assessments.

While radiological staging can guide decision-making, particularly when signs of obstruction or ascites suggest advanced disease, it often fails to fully capture the extent of peritoneal dissemination, especially involving the small intestine. Laparoscopy provides better visualization, making it valuable when CT scan show borderline PCI-scores [14]. However, non-invasive modalities might be preferable. A Dutch multicentre randomized controlled trial is currently investigating the role of MRI as a preoperative tool for predicting eligibility for definite surgery and its potential to reduce the need for surgical staging [15].

Despite the widespread use of PCI score by Swedish colorectal surgeons, our study found that the PCI score was not recorded in 19 of 32 patients (59 %) during the surgical staging procedure. Additionally, these evaluations were not performed or supervised by a HIPEC specialist, which may have influenced the accuracy of staging. This issue is particularly relevant in less experienced centers, where patients with higher PCI scores may be selected for CRS-HIPEC, potentially leading to suboptimal treatment decisions and influencing the overall outcomes. The lack of standardized PCI documentation in staging may have led to inconsistencies, highlighting the need for training and collaboration in centers with less expertise in peritoneal metastasis management.

The median time interval between the staging procedure to definitive surgery was 52 days. Patients with longer intervals often received neoadjuvant therapy or experienced delays due to holiday variations. A separate analysis revealed that the lead time was shorter when staging laparotomy was performed. We also observed that the reporting of PCI scores were more consistently reported in the later period of the study, suggesting that national educational efforts may have improved over time.

When comparing laparoscopy with laparotomy for preoperative staging, we found that the estimated PCI score reported by laparoscopy was higher at CRS-HIPEC, while laparotomy showed less discrepancy. This may be due to the more extensive abdominal exposure provided by laparotomy. However, laparotomy is often performed for cases with adhesions and is associated with longer recovery compared to diagnostic laparoscopy. Laparotomy may be a more effective staging technique when performed by non-HIPEC surgeons in local hospital settings. Alternatively, the use of hand-port assistance during laparoscopy could improve the accuracy of PCI determination.

One of the challenges identified in our study was the disparity between staged and non-staged patients. This likely reflects the fact that surgical staging is more commonly offered to patients with synchronous peritoneal metastases, often before the initiation of neoadjuvant treatment. A higher PCI-score at definite CRS-HIPEC might also reflect a more aggressive tumor biology or host related factors that trigger an increase in peritoneal dissemination [16]. Consequently, the characteristic biological factors of these patients may impact outcomes in ways that are not fully captured by radiological staging or PCI alone.

Another limitation of surgical staging procedures is the difficulty in adequately visualizing the extent of peritoneal metastases in regions affected by adhesions or large tumour masses. However, laparoscopic evaluation performed at a HIPEC centre may be more effective in selecting patients eligible for CRS-HIPEC; minimizing the risk of incomplete cytoreduction, optimizing patient selection, and reducing delays in initiating palliative oncological therapy for patients with extensive disease [7]. Given that operating resources may limit the availability of laparoscopic evaluation at HIPEC centers, educating surgeons in local and regional hospitals may improve the reliability of local staging procedures.

Concerns about port site metastases after laparoscopic surgery have been raised since the procedure´s introduction, with early studies reporting an incidence of 21 %. Nowadays, current rates are below 1 %, similar to the incidence of wound metastases after open surgery [17]. In our cohort, port site recurrences following laparoscopy was 25 %, while no cases of abdominal wall scar recurrences were observed after laparotomy. The significantly higher risk of port site recurrence after previous surgical staging with laparoscopy in patients with peritoneal metastases underscores the need for awareness of the occurrence of abdominal wall recurrences after surgical staging procedures. This findings emphasize the importance of midline port placements, as these sites can easily excised during CRS-HIPEC, and by evacuation of the intraabdominal gases through the trocars before removal, thereby avoiding tumour cells entrapment in the port sites [18]. The potential mechanisms behind the relatively higher rate of abdominal wall recurrences in staged patients suggests that further investigation is needed to determine whether surgical staging itself increases the risk of recurrence. Future research should explore the impact of different staging methods on postoperative outcomes and pattern of metastasis. Based on our observations, we strongly recommend the removal of abdominal midline scars and port sites in cases where recurrences are suspected, either clinically or radiologically.

The incidence of complications with laparoscopic staging has been reported as low in other studies. Valle et al. used video laparoscopy to stage 197 patients with peritoneal metastases, completing full laparoscopic PCI assessments in 196 cases, with only a 2 % complications rate, such as trocar site infection, diaphragm perforation and intraoperative bleeding. Neither Valle nor Iversen reported port site metastases [6], 7]. In general, most of the staging procedures in our cohort were performed at the referral hospitals, and no serious complications were reported, and neither were information on patients considered ineligible for CRS-HIPEC during the staging procedure. Nonetheless, some studies have confirmed the safety and accuracy of diagnostic laparoscopy for patients with peritoneal metastases [19], [20], [21]. Therefore, our primary aim was to evaluate the benefit of staging laparoscopy and laparotomy in selecting patients eligible for CRS-HIPEC, particularly in assessing small bowel involvement. While the use of a separate surgical staging procedure should be selectively considered, the increased risk of abdominal wall recurrences and potential underestimation of peritoneal metastasis extent, particularly during laparoscopy, must be carefully weighed.

A study by Nunez reported a 34 % port site recurrence rate after staging laparoscopy in patients with peritoneal metastases from gastrointestinal, mesothelial, and gynecological origins prior to CRS-HIPEC. Port site recurrence was independently associated with worse overall survival [22]. While we did not find a negative survival impact from abdominal wall recurrences, factors such as PCI ≥21, open & close procedures, and the presence of signet ring cells were independently associated with worse survival. However, we did not account for potential confounders such as differences in surgical techniques, tumor biology, or postoperative care, which could have influenced outcomes.

Many centers routinely resect abdominal wall or port sites during CRS-HIPEC, regardless of pre- or intraoperative evidence of tumour involvement in the abdominal wall [22]. Based on our findings, we believe that surgeons should be aware of the risk of abdominal wall recurrences when considering diagnostic staging with laparoscopy or laparotomy.

## Limitations

Despite the valuable insights provided by this study, several limitations exist. Its retrospective design, missing data on key variables, unmeasured confounding factors, and the small sample size of the staged group limit the statistical power and generalizability of our findings. Moreover, the PCI score was not recorded in many staging procedures, and factors associated with aggressive disease may have contributed to the increased risk of abdominal wall recurrences in the staged group. Selection bias may have been a factor, as patients who underwent staging likely had a higher disease burden. Future studies should aim to include larger patient cohorts, ideally through multicenter collaboration, to enhance the generalizability of our findings and control for selection bias by using statistical methods like propensity score matching. Although our study did not address postoperative complications, quality of life, or long-term oncological outcomes, these factors are crucial for assessing the clinical benefits of preoperative surgical staging in selecting patients for CRS-HIPEC.

## Conclusions

Our study demonstrates that patients undergoing staging laparoscopy or laparotomy experience no negative impact on overall survival or time to recurrence, although there is an increased risk of abdominal wall recurrences. The higher number of ineligible patients for CRS-HIPEC in the surgical staging group may reflect confounding by indication, with these patients likely having more aggressive tumour biology and higher PCI scores. Imaging-based strategies could offer valuable insights into whether surgical staging is the most effective approach, or if less invasive methods could provide similar diagnostic accuracy with fewer associated risks. Future research should explore these alternative methods to ensure the optimal strategy for staging patients with colorectal peritoneal metastases.
